# Effects of CFTR modulators on serum biomarkers of liver fibrosis in children with cystic fibrosis

**DOI:** 10.1097/HC9.0000000000000010

**Published:** 2023-01-20

**Authors:** Steven Levitte, Yonathan Fuchs, Russell Wise, Zachary M. Sellers

**Affiliations:** 1 Division of Pediatric Gastroenterology, Hepatology, and Nutrition, Stanford University, Palo Alto, California, USA; 2 Department of Pharmacy, Lucile Packard Children’s Hospital Stanford, Palo Alto, California, USA

## Abstract

The cystic fibrosis (CF) transmembrane conductance regulator corrector/potentiator combinations lumacaftor/ivacaftor and elexacaftor/tezacaftor/ivacaftor improve sweat chloride, pulmonary function, and nutrition. Yet it is unclear whether they may also impact the progression of liver fibrosis, which is a substantial source of morbidity and mortality for patients with CF. We conducted a retrospective, single-center analysis of children and adolescents with CF treated with lumacaftor/ivacaftor and/or elexacaftor/tezacaftor/ivacaftor therapy, focusing on alterations in liver function tests and fibrosis indices using previously-established thresholds that corresponded with increased liver elastography. In pairwise comparisons of before and during treatment timepoints, we found that those with CF-associated liver involvement experienced significant decreases in gamma-glutamyl transferase, aspartate aminotransferase-to-platelet index, and gamma-glutamyl transferase-to-platelet ratio while on lumacaftor/ivacaftor. These differences were not observed in patients treated with elexacaftor/tezacaftor/ivacaftor, nor were they observed in patients without underlying CF-associated liver disease. These results provide the first evidence that lumacaftor/ivacaftor may improve liver fibrosis in children and adolescents with CF and suggest it may be beneficial in the treatment of CF-associated liver disease.

## INTRODUCTION

Lumacaftor/ivacaftor and elexacaftor/tezacaftor/ivacaftor are approved in the United States for the treatment of cystic fibrosis (CF) in those with homozygous or compound heterozygous F508del cystic fibrosis transmembrane conductance regulator (CFTR) mutations, respectively. Clinical trials showed that these CFTR corrector/potentiator combination drugs improve pulmonary outcomes^1^,^2^; however, there is little data on the impact of these therapies on liver health. This reflects a major unmet clinical need, as liver complications remain a leading cause of death in patients with CF.^3^ We and others have previously shown that gamma-glutamyl transferase (GGT), platelet levels, and the liver fibrosis indices aspartate aminotransferase-to-platelet ratio index (APRI) and GGT-to-platelet ratio (GPR) correlate with imaging-based markers of liver fibrosis and cirrhosis in CF.^4^–^6^ We used these surrogates for liver fibrosis to evaluate the impact of lumacaftor/ivacaftor and elexacaftor/tezacaftor/ivacaftor on children and adolescents with CF with and without abnormal liver fibrosis indices.

## METHODS

### Human subjects

Retrospective analysis of all patients cared for at a tertiary care CF Foundation-accredited academic Pediatric CF Center between January 1, 2013 and December 1, 2021 was undertaken with Institutional Review Board approval (#11197). Inclusion criteria were: (1) treatment with lumacaftor/ivacaftor or elexacaftor/tezacaftor/ivacaftor for ≥6 months, (2) ≥1 set of liver function tests and platelets prior to and during treatment. We included data from 6 patients who participated in open-label extension studies of NCT02514473 and/or NCT03061331; 1 patient participated in the randomized control trial NCT02514473 with unknown randomization, and was excluded.^7^,^8^ Exclusion criteria included liver transplantation. We separated patients based on “liver involvement” or “no liver involvement” prior to starting lumacaftor/ivacaftor or elexacaftor/tezacaftor/ivacaftor. Liver involvement was defined as APRI ≥0.345 or GPR ≥0.403 on at least 2 occasions at least 3 months apart prior to starting CFTR modulator therapy. These cut-offs were previously validated to detect liver fibrosis in CF using ultrasound and magnetic resonance elastography.^4^,^9^

### Liver function tests and liver fibrosis indices

All available laboratory tests were examined during the aforementioned period with focused attention on AST, alanine aminotransferase (ALT), GGT, and platelets. For pretreatment, those labs closest to and before the start of lumacaftor/ivacaftor were used. For post-treatment the most recent labs were used for analysis, with the exception of seven patients who were treated with lumacaftor/ivacaftor followed by elexacaftor/tezacaftor/ivacaftor. For these patients, laboratory values while on lumacaftor/ivacaftor were examined as close as possible to the treatment time on elexacaftor/tezacaftor/ivacaftor. Upper limits of normal (ULN) for AST, ALT, and GGT values were based on Canadian Laboratory Initiative on Pediatric Reference Intervals (CALIPER), which accounts for sex and age.^10^ A platelet value of 150×10^3^/μL was used as the lower limit of normal. Laboratory values were excluded if associated with inpatient admissions, documented infections, or were deemed secondary to other drug treatments based on clinical documentation review by a physician (S.L.) and a pharmacist (R.W.). All laboratory values >2 times the ULN were evaluated to determine if they occurred within 3 months of the patient taking any of the following medications: ciprofloxacin, clarithromycin, erythromycin, ethambutol, fluconazole, itraconazole, levofloxacin, minocycline, posaconazole, and voriconazole. More extensive temporal analysis was performed with values >3 times the ULN, which identified drug-related elevations secondary to amoxicillin/clavulanic acid, sulfamethoxazole/trimethoprim, and tobramycin. APRI and GPR were calculated using CALIPER ULN values using the following equation:


$$\frac{\left[\frac{\left(\mathrm{AST or GGT}\right)}{\left(\mathrm{AST or GGTULN}\right)}\right]}{\mathrm{Platelet count}}\times 100$$

### Statistics

Mean, median, and SD were calculated. Graphs were generated using Prism9 (GraphPad Software, San Diego, CA). Significance was determined by *p* values <0.05 using 2-tailed paired nonparametric Wilcoxon *t* test for pretreatment and post-treatment analysis. χ^2^ test was used to evaluate differences in proportions of patients where indicated.

## RESULTS

### Cohorts

Of the approximately 230 children and adolescents at our Pediatric CF Center we identified 42 patients that were treated with lumacaftor/ivacaftor and 52 that were treated with elexacaftor/tezacaftor/ivacaftor for ≥6 months. Ten patients were excluded due to unavailable laboratory values, resulting in 84 patients (35 lumacaftor/ivacaftor, 49 elexacaftor/tezacaftor/ivacaftor) for analysis. Thirteen of 35 in the lumacaftor/ivacaftor group and 15/49 in the elexacaftor/tezacaftor/ivacaftor group had liver involvement prior to starting CFTR modulator therapy (Table 1). Among these, 7 patients were treated with lumacaftor/ivacaftor followed by elexacaftor/lumacaftor/ivacaftor and had liver involvement before starting lumacaftor/ivacaftor (Table 1).

**TABLE 1:** Characteristics of cohorts

	Lumacaftor/Ivacaftor		Elexacaftor/Tezacaftor/ivacaftor		Lumacaftor/Ivacaftor→Elexacaftor/Tezacaftor/Ivacaftor	
	No Liver Involvement (n=22)	With Liver Involvement (n=13)	No Liver Involvement (n=34)	With Liver Involvement (n=15)	With Liver Involvement (n=7)	
Sex (F:M)	11:11	5:8	19:15	7:8	3:4	
Genotypes, n (%)						
F508del/F508del	22 (100)	12 (100)	17 (50)	9 (60)	7 (100)	
F508del/G542X	0	0	3 (9)	1 (7)	0	
F508del/2183AA->G	0	0	2 (6)	0	0	
F508del/other	0	0	12 (35)	5 (33)	0	
Age at start of CFTR modulator (y)	9.0±4.3	8.8±4.5	15.3±2.6	15.2±2.7	11.7±3.0	15.7±3.1
Time on CFTR modulator (mo)	39.3±13.9	33.3±12.2	16.5±4.3	14.1±4.9	13.1±4.2	14.3±4.8
Percent on prior CFTR modulator, n (%)	0	0	20 (59)	9 (60)	0	7 (100)
On ursodiol, n (%)	—	4 (33)	—	5 (33)	4 (57)	4 (57)
Change in FEV_1_ (Δ% predicted)	−5.4±10.5^a^	+0.5±7.5	+5.4±22.4^b^	+8.7±13.2^c^	−1.7±6.1	+8.0±5.6^d^
Change in BMI (Δ%tile)	+3.6±21.6	−4.8±28.9	+2.9±21.2	+5.8±16.2	−15.8±24.2^e^	+5.3±16.2

FEV1 and BMI were assessed before and during treatment with each CFTR modulator, and compared pairwise using Wilcoxon matched-pairs test.

Abbreviations: BMI indicates body mass index; CFTR, cystic fibrosis transmembrane conductance regulator; F, female; FEV_1_, forced expiratory volume in first second; M, male.

^a^ *p*=0.04.

^b^ *p*<0.001.

^c^ *p*=0.008.

^d^ *p*=0.02.

^e^ *p*=0.03.

### Liver function tests and fibrosis indices in those with and without pre-existing liver abnormalities

**FIGURE 1:**
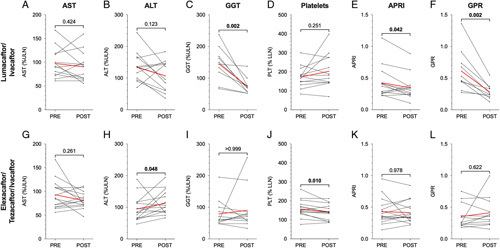
Changes in liver function tests and liver fibrosis indices during lumacaftor/ivacaftor or elexacaftor/tezacaftor/ivacaftor treatment in patients with cystic fibrosis (CF)-associated liver involvement. Single measures of aspartate aminotransferase (AST) (A and G), alanine aminotransferase (ALT) (B and H), gamma-glutamyl transferase (GGT) (C and I), platelets (D and J), aspartate aminotransferase-to-platelet index (APRI) (E and K), and gamma-glutamyl transferase-to-platelet ratio (GPR) (F and L) before and during treatment with cystic fibrosis transmembrane conductance regulator (CFTR) modulator therapy. CF-associated liver involvement was defined as having APRI ≥0.345 or GPR ≥0.403 on at least 2 occasions at least 3 months apart prior to starting CFTR modulator therapy. Individual laboratory values were analyzed and plotted as black circles. Individual patient trends are shown in black lines, and cohort averages are shown in red lines. *p* values are indicated for pairwise comparisons of means.

In order to determine if lumacaftor/ivacaftor and/or elexacaftor/tezacaftor/ivacaftor improves liver outcomes in CF, we examined LFTs and liver fibrosis indices separately in those patients with and without CF-associated liver involvement. We compared values obtained from the same patients prior to starting lumacaftor/ivacaftor with laboratory values obtained after an average (SD) of 33.3±12.2 months of therapy for patients with liver involvement and 39.3±13.9 months in patients without liver involvement. In patients with underlying liver involvement, we observed a significant decrease in GGT, APRI, and GPR (*p*=0.002, 0.042, 0.002, respectively, Figures 1C, E, and F). We did not observe significant differences in AST, ALT, or platelets (Figures 1A, B, and D). Separately, we performed these same analyses on patients who did not have documented liver involvement prior to starting lumacaftor/ivacaftor. We did not observe a significant difference in any of the LFTs or liver fibrosis indices (Supplemental Figures 1A–F, http://links.lww.com/HC9/A35). We performed the same analysis in patients treated with elexacaftor/tezacaftor/ivacaftor [average (SD) of 14.1±4.9 months for those with liver involvement and 16.5±4.3 months for those without liver involvement]. In those with liver involvement, we observed an increase in ALT (*p*=0.048, Figure 1H) and a decrease in platelets (*p*=0.010, Figure 1J). We did not observe significant alterations in AST, GGT, APRI, or GPR (Figures 1G, I, K, and L). In patients without liver involvement treated with elexacaftor/tezacaftor/ivacaftor, we observed increases in ALT, APRI, and GPR (*p*=0.005, 0.027, and 0.023, respectively, Supplemental Figures 1H, 1K, and 1L, http://links.lww.com/HC9/A35) and a decrease in platelets (*p*=0.002, Supplemental Figure 1J, http://links.lww.com/HC9/A35). GGT trended towards an increase although this was not significant (*p*=0.063, Supplemental Figure 1I, http://links.lww.com/HC9/A35).

### Subanalysis of patients with pre-existing liver involvement treated with elexacaftor/texacaftor/ivacaftor

**FIGURE 2:**
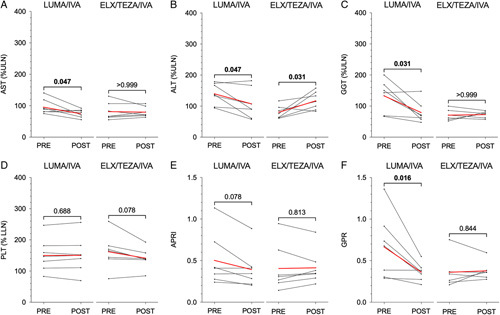
Changes in liver function tests and liver fibrosis indices during lumacaftor/ivacaftor (LUMA/IVA) followed by elexacaftor/tezacaftor/ivacaftor (ELEX/TEZA/IVA) treatment in cystic fibrosis (CF) patients with liver involvement. Aspartate aminotransferase (AST) (A), alanine aminotransferase (ALT) (B), gamma-glutamyl transferase (GGT) (C), and platelets (D) were measured, and aspartate aminotransferase-to-platelet index (APRI) (E) and gamma-glutamyl transferase-to-platelet ratio (GPR) (F) were calculated in the same patients before and during treatment with each cystic fibrosis transmembrane conductance regulator (CFTR) modulator therapy. Individual laboratory values were analyzed and plotted as black circles. Individual patient trends are shown in black lines, and cohort averages are shown in red lines. *p* values are indicated for pairwise comparisons of means.

We next sought to understand why we observed improved GGT, APRI, and GPR in those patients with underlying liver involvement treated with lumacaftor/ivacaftor but not elexacaftor/tezacaftor/ivacaftor. We examined the laboratory values of seven patients treated with lumacaftor/ivacaftor who were subsequently treated with elexacaftor/tezacaftor/ivacaftor and who had evidence of liver involvement before starting lumacaftor/ivacaftor. To first determine if it was related to the longer duration of treatment with lumacaftor/ivacaftor, we controlled for the treatment time with each drug by analyzing laboratory values obtained ∼12 months after starting each therapy [average (SD) 13.1±4.2 months for lumacaftor/ivacaftor vs. 14.3±4.8 months for elexacaftor/tezacaftor/ivacaftor]. While these patients showed improvement in AST, ALT, GGT, and GPRI on lumacaftor/ivacaftor, similar to the liver involvement cohort in (Figure 1), they did not show any further improvements on elexacaftor/tezacaftor/ivacaftor (Figure 2). In fact, ALT worsened with elexacaftor/tezacaftor/ivacaftor. Subanalysis of patients with underlying liver involvement who did not receive prior modulator therapy before starting elexacaftor/tezacaftor/ivacaftor (n=6) did not identify changes in LFTs or liver fibrosis indices aside from a decrease in platelets (*p*=0.030, Supplemental Figure 2, http://links.lww.com/HC9/A36). Together, these results suggest that while lumacaftor/ivacaftor improves surrogate markers of liver injury in patients with underlying evidence of involvement, elexacaftor/tezacaftor/ivacaftor does not appear to have the same effect, at least within the timeframe studied in this patient cohort.

## DISCUSSION

With lung failure the primary cause for mortality in CF, clinical trials examining lumacaftor/ivacaftor and elexacaftor/tezacaftor/ivacaftor focused on changes in pulmonary function.^1^,^2^ However, cirrhosis with portal hypertension is a significant contributor to both morbidity and mortality in CF, especially in children where its peak onset occurs. Because patients with liver disease were excluded from lumacaftor/ivacaftor and elexacaftor/tezacaftor/ivacaftor clinical trials, data on the impact of these drugs on liver health is scarce. Our observations that lumacaftor/ivacaftor improves LFTs and liver fibrosis indices in CF agree with a recent single-center study by Drummond et al.^11^ Of note, neither of these cohorts was examined prior to starting therapy with ultrasound or MRI elastography, which is increasingly being used in conjunction with LFTs to noninvasively quantify fibrosis. In our large academic CF center, the use of ultrasound was not routinely performed for liver disease screening at the time of lumacaftor/ivacaftor approval. While it was being routinely performed prior to elexacaftor/tezacaftor/ivacaftor approval, the COVID-19 pandemic prevented many individuals from receiving a routine ultrasound prior to starting elexacaftor/tezacaftor/ivacaftor. With these caveats in mind, both cohorts likely consisted of those with mild-moderate liver involvement as assessed by biomarkers of fibrosis which correlate well with imaging findings.^9^ It is unclear if our findings are limited to those without advanced liver cirrhosis and/or portal hypertension, but the greatest opportunity to reverse disease likely lies prior to the development of multilobular cirrhosis.

To date, ours is the first study to report on the potential impact of elexacaftor/tezacaftor/ivacaftor on liver fibrosis indices. Given its greater efficacy to improve lung function compared to lumacaftor/ivacaftor, we hypothesized that we may see even better improvement in liver biomarkers with elexacaftor/tezafactor/ivacaftor than lumacaftor/ivacaftor. But we did not. In our liver involvement cohorts, both FEV1 and BMI percentile improved during treatment with elexacaftor/tezacaftor/ivacaftor, arguing that the lack of improvement in LFTs was not due to treatment failure or substantial medication noncompliance. Since all but one patient on lumacaftor/ivacaftor had normalization of GGT and GPR, sensitive markers of early liver fibrosis,^9^ this may have obscured our ability to detect any subsequent change with elexacaftor/tezacaftor/ivacaftor. The PROMISE study (Prospective Study to Evaluate Biological and Clinical Effects of Significantly Corrected CFTR Function) includes evaluation of liver function on elexacaftor/tezacaftor/ivacaftor and may provide further clarity on this question. Our data suggest it may be important to consider prior CFTR modulator exposure when analyzing the PROMISE data.

In summary, we have shown that lumacaftor/ivacaftor improves liver function studies and liver fibrosis indices in patients with pre-existing liver abnormalities. Lumacaftor/ivacaftor may represent the first drug to reverse CF-associated liver fibrosis, but larger multicenter studies with imaging and/or histology are required to validate these findings.

## Supplementary Material

## References

[1] Wainwright CE Elborn JS Ramsey BW Marigowda G Huang X Cipolli M . Lumacaftor-ivacaftor in patients with cystic fibrosis homozygous for Phe508del CFTR. N Engl J Med. 2015;373:220–31. 25981758 10.1056/NEJMoa1409547 PMC4764353

[2] Middleton PG Mall MA Dřevínek P Lands LC McKone EF Polineni D . Elexacaftor-tezacaftor-ivacaftor for cystic fibrosis with a single Phe508del allele. N Engl J Med. 2019;381:1809–19. 31697873 10.1056/NEJMoa1908639 PMC7282384

[3] Toledano MB Mukherjee SK Howell J Westaby D Khan SA Bilton D . The emerging burden of liver disease in cystic fibrosis patients: a UK nationwide study. PLoS One. 2019;14:e0212779. 30947265 10.1371/journal.pone.0212779 PMC6448894

[4] Sellers ZM Lee LW Barth RA Milla C . New algorithm for the integration of ultrasound into cystic fibrosis liver disease screening. J Pediatr Gastroenterol Nutr. 2019;69:404–10. 31181020 10.1097/MPG.0000000000002412

[5] Bodewes FA van der Doef HP Houwen RH Verkade HJ . Increase of serum gamma-glutamyltransferase associated with development of cirrhotic cystic fibrosis liver disease. J Pediatr Gastroenterol Nutr. 2015;61:113–8. 25658056 10.1097/MPG.0000000000000758

[6] Leung DH Khan M Minard CG Guffey D Ramm LE Clouston AD . Aspartate aminotransferase to platelet ratio and fibrosis-4 as biomarkers in biopsy-validated pediatric cystic fibrosis liver disease. Hepatology. 2015;62:1576–83. 26223427 10.1002/hep.28016

[7] (US) NLoM. A study to evaluate the efficacy and safety of lumacaftor in combination with ivacaftor in subjects with CF, homozygous for the F508del-CFTR mutation. In: 8/3/2015-10/23/2017. p. NCT02514473.

[8] (US) NLoM. Lumacaftor/ivacaftor combination therapy in subjects with CF who have an A455E CFTR mutation. In: 2/23/17-10/2/18. p. NCT03061331.

[9] Levitte S Lee LW Isaacson J Zucker EJ Milla C Barth RA . Clinical use of shear-wave elastography for detecting liver fibrosis in children and adolescents with cystic fibrosis. Pediatr Radiol. 2021;51:1369–1377. 33759025 10.1007/s00247-021-05015-w PMC8823212

[10] Estey MP Cohen AH Colantonio DA Chan MK Marvasti TB Randell E . CLSI-based transference of the CALIPER database of pediatric reference intervals from Abbott to Beckman, Ortho, Roche and Siemens Clinical Chemistry Assays: direct validation using reference samples from the CALIPER cohort. Clin Biochem. 2013;46:1197–219. 23578738 10.1016/j.clinbiochem.2013.04.001

[11] Drummond D Dana J Berteloot L Schneider-Futschik EK Chedevergne F Bailly-Botuha C . Lumacaftor-ivacaftor effects on cystic fibrosis-related liver involvement in adolescents with homozygous F508 del-CFTR. J Cyst Fibros. 2022;21:212–219. 34454846 10.1016/j.jcf.2021.07.018

