# Supplementary figures and images for: Effects of CFTR modulators on serum biomarkers of liver fibrosis in children with cystic fibrosis
Source: Hepatol Commun. 2023 Jan 20;7(2):e0010. doi: 10.1097/HC9.0000000000000010 (PMC10019140; doi:10.1097/HC9.0000000000000010)

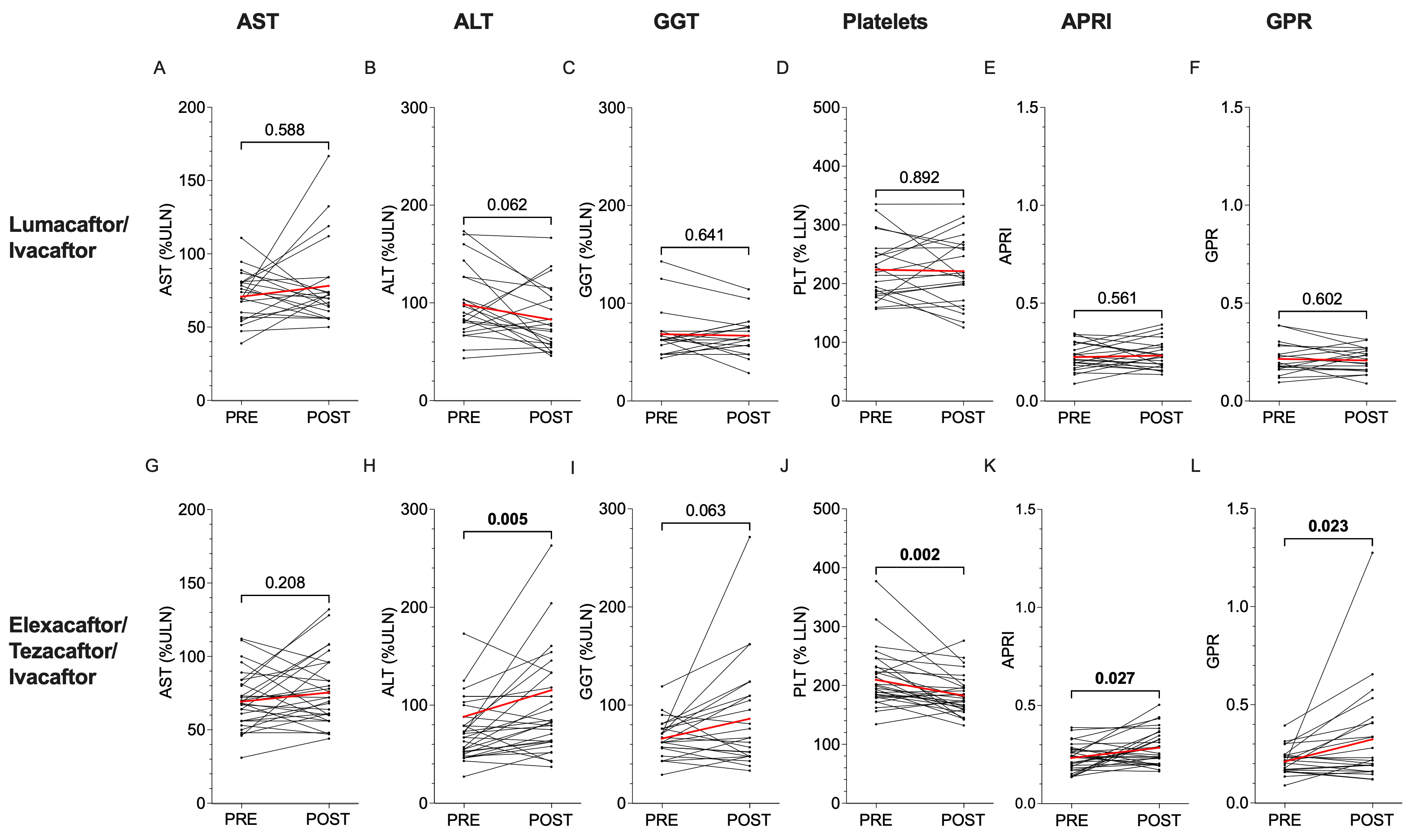

Supplement: Supplementary file 1 [file hc9-7-e0010-s001.tiff]

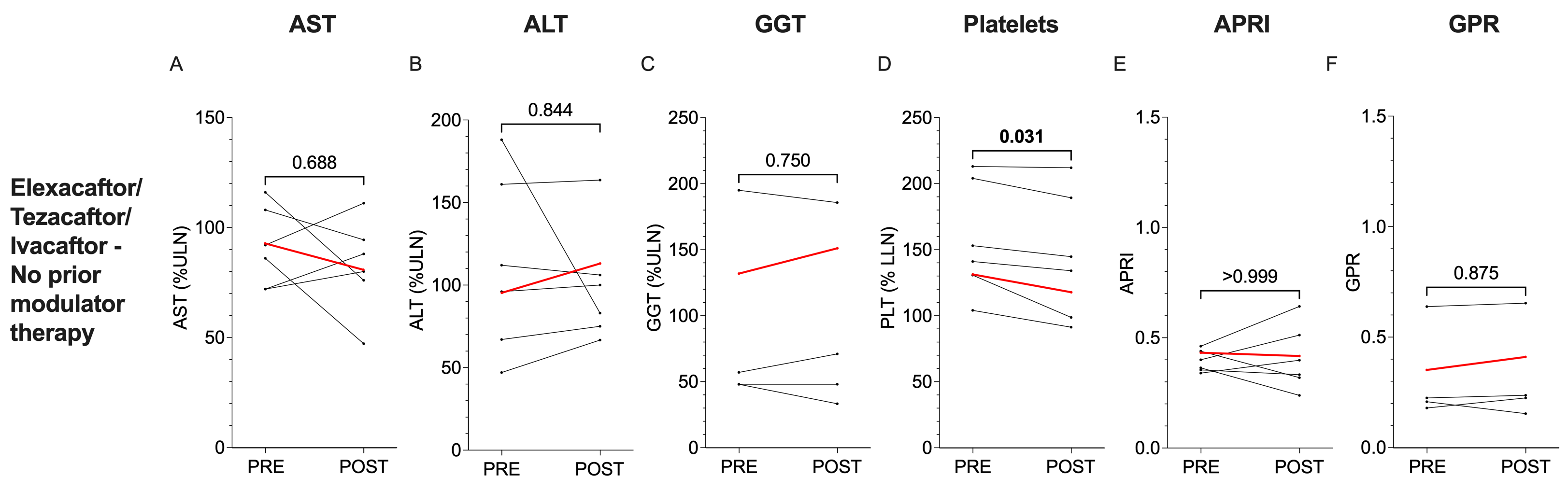

Supplement: Supplementary file 2 [file hc9-7-e0010-s002.tiff]
